# Enhancing cognitive-behavioural therapy for recurrent headache: design of a randomised controlled trial

**DOI:** 10.1186/s12883-014-0233-9

**Published:** 2014-12-11

**Authors:** Paul R Martin, Sharon Mackenzie, Siavash Bandarian-Balooch, Arissa Brunelli, Simon Broadley, John Reece, Peter J Goadsby

**Affiliations:** School of Applied Psychology and Behavioural Basis of Health, Griffith University, Gold Coast Campus, Parklands Drive, Southport, Queensland 4222 Australia; School of Medicine, Griffith University, Gold Coast Campus, Parklands Drive, Southport, Queensland 4222 Australia; School of Health Sciences, RMIT University, PO Box 71, Bundoora, Victoria 3083 Australia; Australian College of Applied Psychology, Melbourne, Australia; Headache Group, NIHR-Welcome Trust Clinical Research Facility, King’s College London, London, UK

**Keywords:** Headache, Migraine, Cognitive behaviour therapy, Coping, Desensitisation

## Abstract

**Background:**

We have argued against the traditional approach of counselling avoidance of all triggers of headaches and migraine. Problems with this approach include the impossibility of avoiding all triggers and the high costs associated with trying to do so, and that avoidance could lead to reduced tolerance for the triggers. We have developed an alternative approach called *Learning to Cope with Triggers* (LCT) that encourages avoidance of triggers that are detrimental to health and wellbeing, but uses exposure to other triggers to desensitise headache sufferers to the triggers. This approach has been shown to be more effective than advising avoidance of all triggers. Trigger management is only one component of a comprehensive treatment program and the current study is designed to evaluate a new approach to treating headaches in which LCT has been integrated into an established cognitive-behavioural therapy (CBT) package (LCT/CBT).

**Methods/Design:**

A target sample of 120 adult participants who suffer from migraine or tension-type headache, at least six days per month, and have done so for at least 12 months will be recruited. Participants will be randomly assigned to one of three groups: LCT/CBT; Avoid/CBT (CBT combined with instructions to avoid all triggers); and waiting-list control. Measures will include: daily diaries for recording headaches, triggers and medication consumption; headache disability and quality of life; trigger avoidance; locus of control and self-efficacy; and coping strategies. Treatment will involve 12 60-minute sessions scheduled weekly. Assessment will be completed before and after treatment, and at 4 and 12 month follow-up. The data will be analysed to determine which approach is most effective, and predictors of response to treatment.

**Discussion:**

Migraine and tension-type headache are common and can be disabling. CBT has been demonstrated to be an efficacious treatment for both disorders. However, there is room for improvement. This study aims to increase the efficacy of behavioural approaches and identify factors predictive of a positive response.

**Trial registration:**

Australian and New Zealand Clinical Trials Registry ACTRN12614000435684.

## Background

It has been estimated that globally, the percentage of the adult population with an active headache disorder is 46% for headache in general, 11% for migraine, 42% for tension-type headache, and 3% for chronic daily headache [1]. In the World Health Organization (WHO) ranking of causes of disability, headache disorders are in the 10 most disabling conditions for both genders, and the 5 most disabling conditions for women. Migraine has been ranked among the diseases causing the greatest degree of handicap, together with conditions such as quadriplegia, dementia and active psychosis [2].

A recent large study of the triggers of migraine attacks found 76% of migraineurs reported triggers when asked, and this figure rose to 95% when individuals responded to a specific list of triggers [3]. The most common headache triggers are: (i) stress and negative emotions; (ii) sensory triggers (flicker, glare, eyestrain, noise, odours); (iii) hunger; (iv) lack of sleep or excess of sleep; (v) food (particularly chocolate, cheese) and drink; (vi) alcohol; (vii) menstruation; and (viii) weather (cold, heat, high humidity) [4-6]. Many other factors have been noted including exercise, fatigue, sexual activity, and head and neck movement. A number of studies have investigated whether migraine and tension-type headache have the same or different triggers, and most have failed to find differences [7-9]. Some more recent studies have found differences with light, odours, hunger, weather and smoke being reported more commonly in migraine than tension-type headache, and head and neck movement being more common in tension-type headache [10,11].

Advice to identify and avoid triggers as a good means of preventing headaches, has been standard practice for decades. Researchers regularly make this point, for example, “comprehensive migraine treatment programs emphasize awareness and avoidance of trigger factors as part of the therapeutic regimen” [12]. One of the ‘seven elements of good headache management’ listed by WHO is “identification of predisposing and/or trigger factors and their avoidance through appropriate lifestyle change” [13]. This advice appears on numerous internet sites. For example, the American Headache Society website includes a handout entitled ‘Trigger Avoidance Information’. Headache Apps are now available for iPhones and iPads that encourage trigger avoidance (e.g., *iManage Migraine*, Merck & Co).

We have published three recent reviews arguing against counselling avoidance of all headache triggers [14-16]. It is not possible to completely avoid all potential headache triggers as they are so diverse; and attempting to do so could result in a restricted lifestyle [3]. It has been pointed out that the effort to avoid every potential headache trigger may itself be stressful [17]. Furthermore, advice to avoid triggers may lead to reduced internal locus of control for headaches, with attendant adverse effects on self-efficacy, particularly concerning perceived capacity to cope effectively with triggers [18].

In the chronic pain literature, fear-avoidance models have been developed, which contend that individuals who confront their pain are considered more likely to adaptively resume physical and social activities, whereas those who respond to pain with anxiety and avoidance are considered more likely to enter a self-perpetuating vicious cycle that maintains and exacerbates pain perception, leading to chronic pain and related disability [19]. In the stress literature, it has been argued that research findings indicate that coping with stress generally takes one of two routes, avoidance or approach, and the evidence demonstrates that the avoidance coping pathway is not adaptive, with a few important exceptions [20]. It has also been argued that higher levels of ‘experiential avoidance’, a type of avoidant coping, are associated with higher levels of general psychopathology and a lower quality of life [21].

The anxiety literature has demonstrated that short exposure to anxiety-provoking stimuli results in increased subsequent anxiety responses to the stimuli, whilst prolonged exposure results in decreased subsequent anxiety responses [22]. It is short exposure, resulting from attempts to avoid, or escape from, anxiety-eliciting situations, that underlies the maintenance of fears and phobias. In contrast, exposure-based approaches have been used with great success to treat a wide range of anxiety disorders [23]. A unified treatment approach for emotional disorders which includes preventing emotional avoidance (including behavioural avoidance and cognitive avoidance) and facilitating emotional exposure has been proposed [24].

A series of studies has investigated the relationship between length of exposure to various headache triggers and the capacity of the trigger to elicit head pain. In participants exposed to the experimentally-validated trigger of ‘visual disturbance’ [25] for one of five durations (‘none’, ‘very short’, ‘short’, ‘long’ and ‘very long’), nociceptive response was greater for the ‘short’ exposure condition than the ‘none’ and ‘very short’ exposure conditions; but the nociceptive response in the ‘very long’ condition was less than in the ‘short’ condition [26]. In summary, the results were consistent with the anxiety literature in that short exposure increased nociceptive response whereas very long exposure decreased nociceptive response. The study was repeated for the validated headache triggers of noise [27] and stress [28] with similar results. In a study in which migraine and tension-type headache sufferers attended the laboratory for six sessions of exposure to visual disturbance, ratings of visual disturbance, negative affect and headache intensity in response to the trigger, decreased from baseline by 44%, 54% and 63%, respectively, demonstrating desensitisation [29].

The arguments against advising avoidance of all headache triggers, and the findings linking prolonged exposure to decreased trigger potency, have led to us developing an alternative approach to trigger management called *Learning to Cope with Triggers* (LCT). The word ‘cope’ is used because of the insights that can be derived from the stress literature which demonstrate that no single coping strategy can be selected as the best way of coping with stress for all situations and across time, but reviewers have concluded that approach strategies generally are more adaptive than avoidance strategies [30]. We have argued that similarly, no one strategy can be singled out as the best way of managing all headache triggers. Sometimes avoidance will be the strategy of choice but more often, approach/engagement/exposure strategies will be more effective.

Following publication of three reviews advocating the LCT approach to trigger management, advice in the literature is now changing [31-33]. For example, the European Federation of Neurological Societies guidelines on the treatment of tension-type headache include “Identification of trigger factors should be performed, as coping with trigger factors may be of value” [31].

We recently published a study designed to evaluate the traditional advice to headache sufferers to avoid all triggers (‘Avoidance’) on the one hand, and LCT that included graduated exposure to selected triggers to promote desensitisation on the other hand [34]. Individuals (84 female, 43 male) with migraine and/or tension-type headache were assigned randomly to one of four groups: Waiting-list (Waitlist); Avoidance; Avoidance combined with CBT (Avoid + CBT); and LCT. Changes in headaches and medication consumption (in parentheses) from pre- to post-treatment were (a minus sign indicates improvement): Waitlist, +11.0% (+15.4%); Avoidance, −13.2% (−9.0%); Avoid + CBT, −30.0% (−19.4%); and LCT, −35.9% (−27.9%). Avoidance did not statistically differ significantly from Waitlist on headaches or medication use, but LCT differed significantly from Waitlist on both measures. Avoid + CBT significantly differed from Waitlist on headaches but not medication consumption. In summary, the study failed to find support for the traditional approach to trigger management of advising avoidance, but LCT emerged as a promising strategy.

Trigger management is only one aspect of a comprehensive approach to the treatment of headaches. Behavioural interventions have been shown to be quite efficacious for the treatment of headaches. A summary of meta-analytic reviews for behavioural treatment of migraine (thermal biofeedback, electromyographic - EMG - biofeedback, CBT, relaxation training) and tension-type headache (EMG biofeedback, CBT, relaxation training) concluded that average improvement ranged from 33% to 55%, compared with 5% for no-treatment controls for migraine, and from 35% to 55%, compared with 2% for no-treatment controls for tension-type headache [35]. We evaluated our version of CBT for migraine and tension-type headache in a randomised controlled trial and found no differences as a function of diagnosis [36]. CBT was associated with the following changes: (i) average decrease in headaches of 68% post-treatment, and 77% at 12-month follow-up; and (ii) average decrease in medication of 70% post-treatment. The average decrease in headaches at post-treatment of 68% (95% CI, 46.44 – 89.56) compares with the range of 33% to 55% from the review [35].

This study will integrate LCT into our version of CBT (LCT/CBT), and evaluate the efficacy of this new approach in anticipation of enhancing the effectiveness of CBT. LCT/CBT will be compared with CBT combined with the standard approach to trigger management of avoidance (Avoid/CBT), and with Waiting-list control (WL). The study will test the following hypotheses:

1. LCT/CBT will result in greater decreases on the primary outcome measures of headaches, medication consumption and headache disability, than Avoid/CBT and WL.
2. LCT/CBT will result in greater increases on the secondary outcome measures of self-efficacy, internal locus of control, and quality of life (less restricted lifestyle), than Avoid/CBT and WL.

The study will also investigate predictors of response to treatment and explore client-treatment matching hypotheses, such as whether participants who have a small number of triggers all of which are avoidable, would respond better to Avoid/CBT than LCT/CBT, whilst the reverse would be the case for participants who have more or less avoidable triggers. The response of migraine versus tension-type headache will be investigated, but there are no specific predictions related to diagnosis as the triggers of the two types of headaches are similar although not identical [10,11].

## Methods/Design

### Participants

#### Inclusion criteria

(i) Diagnosed as either ‘migraine without aura’, ‘typical aura with migraine headache’, ‘chronic migraine’, ‘frequent episodic tension-type headache’, or ‘chronic tension-type headache’; (ii) minimum of 6 headache days per month; (iii) minimum headache chronicity of 12 months, and pattern of headache symptoms stable over last six months; (iv) medication use stable for a minimum of one month; and (v) aged 18 to 75 years.

#### Exclusion criteria

(i) Diagnosed as ‘medication-overuse headache’; (ii) headaches present continuously; (iii) undergoing behavioural treatment; and (iv) substantial medical or psychiatric comorbidities that are deemed likely to interfere with ability to fully participate.

All diagnoses will be based on The International Classification of Headache Disorders, 3rd edition (beta version) (ICHD-3 beta) [37].

#### Recruitment

The target sample size is 120. A range of recruitment strategies will be used as in our previous headache research including: general practitioner (GP) referrals (targeting practices directly and through the Divisions of General Practice); the media (television, community and national newspapers, radio); posters; organisations that have agreed to assist using their websites or newsletters (e.g., Headache Australia, Brain Foundation); intranet (Griffith University, Gold Coast Health); and internet, podcasts, YouTube and Facebook.

### Research design

The design is a mixed design, consisting of a between subjects factor (Group), and a within subjects factor (Time). A computer-generated sequence will be used, and the Consolidated Standards of Reporting Trials (CONSORT) guidelines followed for the randomisation procedure. The author with statistical responsibility and no involvement with participants (JR), will take responsibility for the randomisation. The study is a single-site (university setting), single-blind, Phase II clinical trial. The study is being conducted in compliance with the Helsinki Declaration and has been approved by the Griffith University Human Research Ethics Committee (GU Ref No: PSY/31/13/HREC). Written informed consent is required for all participants.

### Measures

#### Demographic information, headache diagnosis and functional assessment

We have developed and used in previous studies e.g., [34] the following questionnaires to collect this background information: (i) Personal and Social History Questionnaire (PSHQ); (ii) Headache Diagnosis Questionnaire (HDQ) (used in conjunction with our *Manual for Diagnosing Primary Headache*, based on ICHD-3 beta); and (iii) Functional Assessment of Headache Questionnaire (FAHQ). The PSHQ collects demographic information such as age, gender, marital status, children, educational level, and occupation. The HDQ is a structured diagnostic interview with questions derived directly from ICHD-3 beta. The FAHQ includes questions about the antecedents (immediate, setting, onset and predisposing) and consequences (immediate and long-term for sufferers and significant others) of headaches. The PSHQ and FAHQ are derived from the Psychological Assessment of Headache Questionnaire [38].

#### Avoidance of headache triggers

We will use the Trigger Specific Avoidance Scale of the Headache Triggers Avoidance Questionnaire (HTAQ) (Wood A, Martin PR: *Development of the Headache Triggers Avoidance Questionnaire*, in preparation). This Scale has 24 items and measures how often respondents try to avoid the factors that trigger their headaches. It has good internal consistency (Cronbach alpha of .81), good test-retest reliability over 3 to 4 weeks (*r* = .90, *p* < .001), and construct validity shown via significant correlations with related scales such as the Anxiety Sensitivity Index (*r* = .27, *p* < .001) and Pain Anxiety Symptoms Scale (*r* = .37, *p* = < .001).

#### Self-efficacy

The Headache Management Self-Efficacy Scale (HMSES) consists of 25 items that inquire about an individual’s confidence in her/his ability to prevent and to manage headaches [39]. It has good internal consistency and construct validity.

#### Locus of control

The Headache-Specific Locus of Control Scale (HSLC) consists of 33 items for which participants indicate the degree to which they believe that the variables controlling headache activity are primarily internal or external [40]. It has three subscales (healthcare professional, internal, chance), all with good test-retest reliability and construct validity.

#### Coping strategies

Coping strategies will be assessed with the Coping Strategies Inventory (CSI) [41,42]. The CSI has 72 items designed to assess coping strategies used in responding to stressful life events, and includes 8 subscales, 4 for ‘engagement’ strategies (e.g., social support), and 4 for ‘disengagement’ strategies (e.g., avoidance). The CSI will be included as we have found the social support subscale to be the best predictor of response to CBT [36].

#### Headaches, triggers and medication

For several decades, headache diaries have been recorded using paper-and-pencil, but such diaries have several important limitations including high researcher effort and error in data entry, high percentage of participant non-compliance, and inaccurately completed and missing items [43,44]. We have developed an e-diary using mobile software technology that can be used on a wide range of smartphone operating systems or installed on Windows computers. Research has shown that computerised pain diaries overcome several limitations of paper diaries [45], and recording exact time and date of diary completion significantly increases compliance rates [46,47]. In accordance with recommendations on the need to reduce client burden, the e-diary only includes seven items, which are widely considered as core questions to include in behavioural headache research [48]. The items are: current headache intensity; peak intensity; average intensity; number of headaches; average duration of headaches; medication consumption (number of pills taken); and headache triggers (checklist of 26 triggers). Participants are required to complete the e-diaries each day just prior to going to sleep, and a paper version is available for those who cannot use the e-diary system.

#### Functional status

Collecting data on measures of disability and quality of life, as well as primary and secondary measures of headache and non-headache measures (e.g., medication consumption) has been recommended [49]. The functional and emotional impact of headache on everyday life will be measured by the Headache Disability Inventory (HDI), and health-related quality of life by the Short-Form Health Survey version 2 (SF-36v2). The HDI is a 25-item questionnaire that has good internal consistency, long-term test-retest reliability and construct validity [50]. The SF-36v2 is a 36-item questionnaire from the Medical Outcomes Study, which measures health-related quality of life in eight areas (e.g., physical functioning, general and mental health).

### Treatment conditions

#### LCT/CBT

A therapist manual and client handbook were developed for a 12-session program of 60-minute sessions scheduled weekly by integrating existing materials. The LCT component of the manual specifies principles for identifying triggers and deciding what strategies to use for each trigger. For example, ‘planned exposure’ may serve three functions: (i) exposure as an ‘experiment’ to see if the alleged trigger does indeed precipitate headaches (e.g., foods are often incorrectly identified as headache triggers [51,52]); (ii) exposure to achieve desensitisation/habituation/adaptation; and (iii) exposure to enable practising coping skills. Exposure is generally viewed as the strategy of choice for triggers such as stress and negative affect, and sensory triggers (e.g., visual disturbance, noise). Avoidance is generally seen as the strategy of choice for triggers that are not consistent with a healthy lifestyle such as toxic smells, hunger, dehydration and lack of sleep. Menstruation is viewed as a cue to focus on the other triggers that often give rise to headaches in combination with hormonal factors. The parameters of exposure to triggers (length of exposure and intensity of trigger stimulus) are always manipulated such that they fall short of precipitating significant headaches. Evidence-based strategies are used whenever possible, such as for stress (e.g., [53]) and anxiety (e.g., [23]).

The CBT component of the program is derived from the book *Psychological Management of Chronic Headaches* [38], and has been shown to be effective [36]. This approach to CBT adopts a functional model that seeks to understand the controlling variables of headaches. It aims to answer questions such as: why does a headache occur at one time rather than another; why is this person experiencing headaches at this point in her/his life rather than at other times; why did the headache problem begin when it did; and why is this person vulnerable to developing a headache disorder? Functional models consider antecedents and consequences in seeking answers to these questions. As the immediate antecedents of headaches are the triggers, LCT integrates easily into this version of CBT. There is much overlap with CBT as used by other research groups, such as including education, challenging dysfunctional thoughts and beliefs, relaxation, pain management and relapse prevention. However, a functional analysis might indicate, for example, that treatment should address the setting antecedents (psychosocial context) for headaches by focussing on the source of stress as a trigger (e.g., dysfunctional marriage) or stress mediating variables (e.g., deficient social support). Alternatively, it might indicate that maladaptive reactions to headaches by the sufferer or their significant others should be part of the treatment program.

#### Avoid/CBT

This condition is a version of the equivalent in the MaTCH study (Avoid + CBT), extended from 8 to 12 sessions [34]. As for LCT/CBT, a therapist manual and client handbook were developed. Sessions will be 60-minutes each and scheduled weekly.

#### WL

Participants in this condition will not receive any treatment until after the ‘post-treatment’ assessment period. They will then be offered LCT/CBT or Avoid/CBT (their choice), which will give the opportunity for further evaluation. It will mean that these participants will be lost to follow-up, but it is considered unethical to withhold treatment for a longer period.

### Therapists

Therapy will be delivered by registered psychologists who have completed postgraduate professional qualifications in clinical or counselling psychology.

### Treatment integrity

**Treatment fidelity** (intervention actually delivered as intended) will be assessed via a combination of therapists completing checklists, and scoring 10% of videos (randomly selected) of treatment sessions [54]. The checking will be carried out blind to the intervention (therefore not by the administering therapist). **Treatment adherence** (patient compliance with procedural requirements) will be assessed via participants self-monitoring [54,55]. Treatment integrity is considered particularly important in this study as the two treatment conditions overlap, with both involving CBT and even the trigger management strategies not being completely different as LCT does not involve promoting approach/engagement strategies rather than avoidance with all triggers. A key target in analysing treatment integrity will be documenting triggers for which approach/engagement versus avoidance strategies are utilised.

### Procedure

#### Pre-treatment assessment and baseline self-monitoring

All individuals volunteering for the study will undergo a telephone screening and those who meet the screening criteria will be invited to an assessment session that takes approximately three hours to complete. At this session, participants will complete informed consent procedures, and the HDQ. They will then complete an electronic survey that includes the SF-36v2, HTAQ, HSLC, HDI, HMSES, and CSI. Next, they will complete the PSHQ, and FAHQ. Participants will then be given training in use of the e-diaries to monitor headaches, triggers and medication consumption. Self-monitoring will take place for four weeks prior to the commencement of treatment.

#### Randomisation

Participants will be randomly allocated to the three groups in the study after the assessment session.

#### Treatment

Participants will receive one of the three ‘treatments’ over the next 12 weeks.

#### Post-treatment assessment

Participants will self- monitor their headaches, triggers, and medication consumption for four weeks after treatment has concluded, and then attend a psychologist, blind to treatment condition, for a clinical assessment as a ‘collateral’ measure of outcome. They will complete the electronic survey with the six questionnaires embedded in it as described above.

#### Four- and 12-month follow-up assessments

Participants will attend a psychologist, blind to treatment condition, 4 and 12 months after the 12-week treatment period. They will be asked to resume self-monitoring headaches, triggers and medication consumption for a 4-week period, and to complete the electronic survey.

#### Quality control

A detailed operations manual has been prepared. The staff have all attended a 7-hour workshop training them in the study procedures, presented by the primary investigator who has over 40 years of experience in research, clinical practice, and training health professionals in the headache domain. Weekly meetings will be scheduled for the primary investigator to provide supervision for the research staff. This will include review of ‘cases’. Data will periodically be forwarded to the investigator with primary responsibility for analysing the data (JR), for review.

### Statistical analyses and power considerations

The study design will focus on the interaction test of a mixed factorial design. Data will be tested for assumptions underlying both univariate and multivariate parametric analysis.

The power analysis is based primarily on the effect sizes generated in previous clinical trials of *f* = 0.33 to 0.42. Based on previous results an effect size of *f* = 0.25 (commonly accepted convention for a medium effect) can be confidently predicted. Power analysis using G*Power 3.1 software package indicates that for a three-group mixed factorial design with 40 participants per group and alpha = .05, moderate effects (*f* = .25) would be reliably identified with power .93 and a large effect (*f* = 0.4) would have an almost certain probability of being reliably identified.

Any missing data will be considered on an intention-to-treat basis [56,57] and fully analysed for the presence of Missing At Random, Missing Completely At Random, and Not Missing At Random patterns [58]. If statistically appropriate, missing data will be imputed using case-level multiple imputation. Data will be tested for assumptions underlying both univariate and multivariate parametric analysis. In addition to exploratory descriptive and correlational analyses, group by phase factorial ANOVAs using a Linear Mixed Models approach will be the main method of analysis for assessing treatment outcome. These will be supplemented by analyses of simple main effects and trend analysis (i.e., analysis of orthogonal polynomials) to examine the pattern of change over time for the experimental groups.

A linear mixed modelling approach has been chosen in order to permit: flexible structuring of the repeated covariance associated with the phase effect; more options for dealing with missing values; the analysis of individual change via growth curves; and options for analysing multi-level models, should they be theoretically or clinically relevant. For all inferential tests, measures of effect size (i.e., either *d* or η^2^) will be calculated, including confidence intervals around those measures. A range of clinical significance measures will also be considered [59]. Finally, regression analyses will be used to examine predictors of response to treatment, and moderation analysis will be used to examine the client-treatment matching hypotheses [60].

## Discussion

Headache disorders are among the most common disorders of the nervous system, causing substantial disability in populations throughout the world [13]. This has resulted in the *Global Campaign against Headache Disorders: “Lifting the Burden”*, launched jointly by WHO, International Headache Society, World Headache Alliance and European Headache Federation [61,62]. On 17th October 2011, in recognition of the importance of headache as a pain disorder, the International Association for the Study of Pain launched the *Global Year Against HEADACHE Oct 2011-Oct 2012.*

An extensive literature has accumulated demonstrating that various behavioural treatments are efficacious for both migraine and tension-type headache. The United States Headache Consortium developed evidence-based guidelines for the treatment of migraine based on an extensive review of the medical literature and compilation of expert consensus, and found Grade A evidence (‘multiple well-designed randomised clinical trials, directly relevant to the recommendation, that yield a consistent pattern of findings’) in support of behavioural treatments (thermal biofeedback, EMG biofeedback, CBT and relaxation) for migraine [63]. Nevertheless, there is room for improvement of behavioural interventions.

The proposed research represents arguably the first attempt to improve the efficacy of CBT for headaches. It does so by incorporating into the approach a method of trigger management that has been shown to be superior to the traditional method of trigger management of encouraging avoidance of all triggers. The proposed research is an efficacy trial and if the results are as predicted, then effectiveness research should follow (how well it works in the field).

## Abbreviations

LCT: Learning to Cope with Triggers
CBT: Cognitive-behavioural therapy
WHO: World Health Organization
EMG: Electromyographic
WL: Waiting-list
ICHD-3 beta: International Classification of Headache Disorders, 3rd edition (beta version)
GP: General practitioner
CONSORT: Consolidated standards of reporting trials
PSHQ: Personal and Social History Questionnaire
HDQ: Headache diagnosis questionnaire
FAHQ: Functional assessment of headache questionnaire
HTAQ: Headache triggers avoidance questionnaire
HMSES: Headache management self-efficacy scale
HSLC: Headache-specific locus of control scale
CSI: Coping strategies inventory
HDI: Headache disability inventory
SF-36v2: Short-Form Health Survey version 2
